# The influence of leukocytospermia on the outcomes of assisted reproductive technology

**DOI:** 10.1186/1477-7827-10-44

**Published:** 2012-06-15

**Authors:** Mario Cavagna, Joao Batista A Oliveira, Claudia G Petersen, Ana L Mauri, Liliane F I Silva, Fabiana C Massaro, Ricardo L R Baruffi, José G Franco

**Affiliations:** 1 Center for Human Reproduction Prof Franco Jr, Ribeirao Preto, Brazil; 2 Women's Health Reference Centre, Hospital Perola Byington, Sao Paulo, Brazil; 3 Paulista Centre for Diagnosis, Research and Training, Ribeirao Preto, Brazil; 4 Department of Gynaecology and Obstetrics, Botucatu Medical School, São Paulo State University - UNESP, Botucatu, Brazil

**Keywords:** Leukocytospermia, ICSI, IMSI, Reactive oxygen species, DNA damage

## Abstract

**Background:**

It is not well established whether the increased number of leukocytes in the seminal fluid impairs the outcomes of assisted reproductive technology (ART). This investigation analysed the outcomes of the intracytoplasmic sperm injection (ICSI) and intracytoplasmic morphologically selected sperm injection (IMSI) cycles in couples in which the male partner exhibited leukocytospermia.

**Methods:**

A total of 100 cycles in 100 couples were included in this study. For the ICSI or IMSI procedures, the patients were divided into two groups according to the presence or absence of leukocytospermia and then matched by (female) age:

ICSI: Group I (n = 25): Leukocytospermia - semen samples with a leukocyte count of greater than or equal to 1 × 10(6)/mL; and Group II (n = 25): Non-leukocytospermia - semen samples with a leukocyte count < 1 × 10(6)/mL.

IMSI: Group I (n = 25): Leukocytospermia; and Group II (n = 25): Non-leukocytospermia.The endpoints included the rates of fertilisation, implantation, clinical pregnancy, miscarriage, ongoing pregnancy and live birth. Student’s t-tests, Mann–Whitney tests and Chi-square tests were performed, and *P* < 0.05 was considered significant.

**Results:**

The data from the ICSI groups showed that leukocytospermia did not have a negative influence on the rates of fertilisation (Group I: 57.9+/−30.2%, Group II: 61.9+/−27.7%; *P* = 0.74), implantation (Group I: 12.3%; Group II: 13.5%; *P* = 0.93), clinical pregnancy (Group I: 24%; Group II: 24%; *P* = 1.0), miscarriage (Group I: 0, Group II: 0), ongoing pregnancy (Group I: 24%; Group II: 24%; *P* = 1.0), or live births (Group I: 24%; Group II: 24%; *P* = 1.0). Similarly, the data from the IMSI groups also showed that the leukocytospermia did not have a negative influence on the rates of fertilisation (Group I: 67.6+/−24.6%, Group II: 59.5+/−28.1%; *P* = 0.36), implantation (Group I: 17.5%; Group II: 16.7%; *P* = 0.90), clinical pregnancy (Group I: 28%; Group II: 24%; *P* = 1.0), miscarriage (Group I: 14.3%; Group II: 0; *P* = 0.33), ongoing pregnancy (Group I: 24%; Group II: 24%; *P* = 1.0), or live births (Group I: 24%, 6/25; Group II: 24%, 6/25; *P* = 1.0).

**Conclusions:**

The results indicate that the leukocytospermia may not have a negative effect on the outcomes of ICSI or IMSI cycles. Nevertheless, it seems that it is necessary to more precisely determine the effects, if any, of seminal leukocytes on fertilisation and implantation processes. Such efforts will help to establish a more reliable leukocyte threshold, which could eventually demonstrate whether there is a negative influence on the ART procedures.

## Background

Male infertility represents an important etiological factor for infertile couples to consider, and semen analysis is the main screening tool that guides diagnosis. Among the parameters evaluated in the seminal analyses, the white blood cell count is considered, as are sperm concentration, motility and morphology. According to the World Health Organization (WHO), a leukocyte count ≥1 × 10^6^/mL in a semen sample is indicative of leukocytospermia [1]. An elevated leukocytes count is found in the seminal fluid of approximately 30% of infertile men, even in the absence of inflammatory symptoms or a seminal bacterial infection [2]. Reports on the influence of leukocytospermia on male fertility are contradictory [3-5]. Nevertheless, there is evidence indicating that leukocytospermia has a negative impact on semen quality due to the production of reactive oxygen species (ROS) [6-8]. Although there is ongoing controversy concerning the effects of leukocytospermia on the integrity of sperm DNA [9], it was recently reported that semen samples with leukocytospermia are more likely to evidence sperm with DNA fragmentation [10,11]. However, few reports have analysed the influence of leukocytospermia on the outcomes of assisted reproductive technology (ART) [12-17].

Some authors have demonstrated the influence of leukocytospermia on *in vitro* fertilization (IVF)/ intracytoplasmic sperm injection (ICSI) outcomes. Using multivariate analysis of the semen parameters in 195 couples undergoing *in vitro* fertilisation, Talbert et al. [12] observed that semen parameters correlating most closely with the reduced ability to fertilise mature oocytes included two primary factors, including a slow rate of forward progression of sperm and the presence of excess numbers of white cells in the semen. Krausz et al. [13] investigated the influence of two factors, including ROS generated by the human spermatozoa and contaminating leukocytes, on sperm movement and *in vitro* fertilisation. The authors showed that the presence of leukocytes was associated with elevated levels of spontaneous ROS production, impaired movement, and a reduced capacity for *in vitro* fertilisation. Sukcharoen et al. [14] placed a particular emphasis on the degree of leukocyte contamination as a criterion that accurately predicts the fertilising potential of human sperm suspensions *in vitro*. The results of the study of Yilmaz et al. [15] indicated that some semen parameters and the outcome of ICSI were negatively affected by the presence of leucocytospermia. Conversely, other authors have questioned the influence of these leukocytes on the results of ART. Lackner et al. [16] found that leukocytospermia has no negative influence on fertilisation or pregnancy rates following either IVF or ICSI; however, one limiting factor of this study was the small number of leukocytospermic semen samples in both the IVF and ICSI groups. Barraud-Lange et al. [17] observed that, at moderate levels (<10^6^/mL), leukocytospermia appears to be within the physiologic range. It is associated with an improved capacity for sperm fertilisation and pregnancy outcomes. At higher concentrations, however, leukocytospermia alters neither sperm fertilisation ability nor the probability of clinical pregnancy, compared with nonleukocytic patients with infertility. However, in the latter case, the pregnancy rate was reduced.

Innovative ART methods developed for the selection of sperm have provided new insight into the interaction between the sperm quality and clinical results [18,19]. To test the hypothesis that subtle sperm organelle malformations are associated with ART results, Bartoov et al. [20,21] developed a method of evaluating human spermatozoa in real time at a high magnification (≥ 6000x), which is called motile sperm organelle morphology examination (MSOME). This method promoted the development of the intracytoplasmic morphologically selected sperm injection (IMSI) technique. IMSI is based on sperm normality, as defined by the MSOME classifications, and it is aimed at improving the conventional ICSI outcomes by focusing on the relation between the sperm morphological abnormalities observable at high magnification and related DNA damage [21-25]. Various studies have demonstrated that IMSI significantly improves embryo quality [21,26], the rate of development up to the blastocyst stage [27,28], the rates of implantation and pregnancy after embryo transfer on days 2 or 3 [21,22,26,29-31] or during the blastocyst stage [28], as well as the likelihood of having a normal, healthy child [32]. IMSI also appears to significantly decrease miscarriage rates [21,22,28,30]. Considering that ROS production, which is potentially associated with the presence of leukocytes in the semen, has been correlated with increased sperm DNA fragmentation [7,9,33], it is plausible that the use of IMSI may benefit the clinical outcomes of patients with leukocytospermia. These benefits would be based on the potential to exclude morphological sperm abnormalities that are potentially related to DNA damage. However, the repercussions of the clinical use of IMSI in patients with leukocytospermia have not yet been determined.

Therefore, this study was designed to assess the effect of leukocytospermia on the clinical outcomes (e.g., the rates of fertilisation, implantation, pregnancy, miscarriage, ongoing pregnancy and live births) associated with ICSI and IMSI.

## Methods

### Study participants

A total of 100 couples who were enrolled in the IVF/ICSI programme of the Centre for Human Reproduction Prof Franco Jr. and who were in their first cycles of treatment were included in this study.

The couples were referred for either the ICSI or the IMSI procedures.

### The ICSI procedure

The patients were divided into two groups according to the presence or absence of the leukocytospermia based on the seminal evaluations and matched by (female) ages: Group I (study, n = 25): Leukocytospermia semen samples with a leukocyte count ≥1 × 10^6^/mL; and Group II (control, n = 25): Non-leukocytospermia semen samples with a leukocyte count <1 × 10^6^/mL.

### The IMSI procedure

The patients were divided into two groups according to the presence or absence of leukocytospermia based on the seminal evaluations and matched by (female) ages: Group I (study, n = 25): Leukocytospermia semen samples with a leukocyte count ≥1 × 10^6^/mL; and Group II (control, n = 25): Non-leukocytospermia semen samples with a leukocyte count <1 × 10^6^/mL.

All of the couples who enrolled in the study met the following inclusion criteria: subfertility with a need for assisted reproduction with ejaculated spermatozoa, no previous ART attempts, a maternal age ≤ 39 years, a normal karyotype in both partners and a lack of uterine defects, ultrasonographic evidence of hydrosalpinx, infections, endocrine problems, coagulation defects or thrombophilia and autoimmune defects (including the presence of anti-phospholipid antibodies). The microbiological analyses of the semen were conducted during routine semen evaluations. Bacterial examinations of the semen were not carried out on the same day as the IMSI or ICSI procedures. The group assignments to the ICSI or IMSI procedures was performed by serial entry, with alternate allocations per set of two treatment cycles (i.e., two allotted to the ICSI procedure then two allotted to the IMSI procedure). The division between the groups was performed as follows: after the referral of a couple whose male partner had leukocytospermia for ICSI or IMSI, the following couple with the same characteristics (i.e., of same age (± 1 year) and fulfilled the inclusion criteria) whose male partner did not have leukocytospermia was referred for either ICSI or IMSI, respectively. In total, 150 couples were screened for this study. The 50 couples whose male partners exhibited no leukocytospermia were not included because they did not share the same characteristics of the couples with leukocytospermia included immediately prior to their assessments. Written informed consents were obtained from all couples, and this study was approved by an internal institutional review board.

### Ovarian stimulation and oocyte recovery culture protocol

All of the patients were submitted to the same protocol of controlled ovarian stimulation. First, the extent of pituitary downregulation was established with leuprolide acetate at a dose of 1 mg/day (Lupron; Abbott, Brazil), which was started during the luteal phase of the previous cycle. After 14 days of treatment with the GnRH analogue and after a confirmation of a pituitary downregulation, the administration of recombinant FSH (Gonal-F®; Serono Barueri, SP, Brazil) was initiated at a dose of 150–225 IU (with doses dependent on the patient’s age) and 75 IU/day of recombinant LH (Luveris®, Serono, SP, Brazil) for a period of 7 days. When one or more follicles measuring ≥17 mm were observed, recombinant HCG (Ovidrel®; Serono, Brazil) was administered at a dose of 250 μg, and oocyte retrieval was scheduled 35–36 hours later.

### Semen preparation

Semen samples were collected in sterile containers via masturbation after a sexual abstinence period of 2 to 5 days. The liquefied, fresh semen samples were prepared using an ISolate discontinuous concentration gradient (Irvine Scientific, Santa Ana, CA, USA). The final semen pellet was resuspended in 0.2 mL of modified HTF medium (Irvine Scientific, Santa Ana, CA, USA) supplemented with 10% human serum albumin (Irvine Scientific, Santa Ana, CA, USA).

Each original/fresh semen sample was analysed for the standard semen quality parameters according to the WHO guidelines [1]. An observation of the presence or absence of leukocytospermia, which was defined as the presence of ≥1 × 10^6^/mL of white blood cells, was carried out at this point. Sperm morphology was analysed according to the MSOME criteria when the couple underwent the IMSI procedure [20].

### ICSI procedure

A conventional ICSI was performed using a Nikon Eclipse TE 300 inverted microscope, equipped with the Narishige 231 D-2 remote-controlled hydraulic micromanipulators (Narishige, Tokyo, Japan) and the Narishige IM-9B injectors. Spermatozoa were selected at 400x magnification using a Hoffman modulation contrast based on a set of published guidelines.

### IMSI procedure

A 1-μL aliquot of sperm cell suspension was transferred to a 5-μL microdroplet of modified HTF medium that contained 7% polyvinylpyrrolidone solution (PVP medium; Irvine Scientific, USA). The microdroplet was placed into a sterile glass dish (FluoroDish^TM^; World Precision Instruments, USA) in sterile paraffin oil (Ovoil-100; Vitrolife, Goteborg, Sweden). Sperm cells that were suspended within the microdroplet were placed on a microscope stage, above an Uplan Apo 100 oil/1.35 objective lens that was previously covered by a droplet of immersion oil. In this manner, the suspended motile sperm cells in the observation droplet could be examined at a high magnification through an inverted microscope (Eclipse TE 2000 U; Nikon, Japan) equipped with high-powered differential interference contrast optics (DIC/Nomarski). Images were captured using a colour video camera that contained effective picture elements (pixels) for the production of high-quality images, and these images were projected onto a colour video monitor. The morphological evaluation was accomplished using a monitor screen, and the total calculated magnification was 8400x (total magnification: objective magnification = 100x, magnification selector = 1.0x, video coupler magnification = 1.0x and calculated video magnification = 84.50x).

The spermatozoon used for IMSI was classified as morphologically normal if it exhibited a normal nucleus, an acrosome, a post-acrosomal lamina, a neck and a tail, and did not present a cytoplasmic droplet or cytoplasm around the head [20]. The subcellular organelles were morphologically classified based on the presence of specific malformations. These malformations were defined according to the subjective descriptive approach reported by Bartoov et al. [20] based on studies utilising transmission and scanning electron microscopy. According to results obtained using transmission electron microscopy [20], a morphologically normal state of the nucleus was defined by the shape and content of the chromatin. The criterion for normality of the nuclear form was a smooth, symmetrical and oval configuration. The normal means for the length and width were estimated as 4.75 ± 2.8 and 3.28 ± 0.20 μm [20], respectively, and the form was classified as abnormal when it presented a variation of ±2 standard deviations on one of its axes (length: ≥5.31 or ≤4.19 μm; width: >3.7 or <2.9 μm). For a rapid evaluation of the nuclear form, a fixed, transparent, celluloid form of a sperm nucleus fitting the criteria was superimposed on the examined cell (chablon construction based on ASTM E 1951-2 [34]). In the same manner, the nuclear form was considered abnormal if an extrusion or invagination of the nuclear chromatin mass was detected (i.e., a regional malformation of the nuclear form). The chromatin content was considered abnormal if one or more vacuoles occupied more than 4% of the nuclear area (if necessary, visual evaluation was aided by superimposing a celluloid form of a large vacuole on the examined cell). A nucleus was considered normal if both the nuclear form and chromatin content were normal.

The same technician performed all sperm selections, and only normal spermatozoa were injected in this study. The time involved in the selection step was 30–60 min/sample, and at least three spermatozoids were selected for each MII oocyte. After the sperm selection process, the IMSI microinjections were conducted in the same manner as for the ICSI injections. The spermatozoids were still motile when captured for the final selection.

### Oocyte and embryo culture and transfer

The sperm-injected oocytes, zygotes and embryos from both of the IMSI (Leukocytospermia and Non- leukocytospermia) and ICSI (Leukocytospermia and Non-leukocytospermia) groups were subjected to the same culture conditions. On day 2, the two to three best-scoring embryos from both of the ICSI (Leukocytospermia and Non-leukocytospermia) and IMSI (Leukocytospermia and Non-leukocytospermia) groups were transferred. The quality of the transferred embryos was similar among the four groups.

### Statistical analysis

The data were analysed using InStat version 3.0 (GraphPad Software, San Diego, California, USA) on a Macintosh computer (Apple Computer Inc., Cupertino, California, USA). The endpoints of the investigation included the rates of fertilisation, implantation, clinical pregnancy, miscarriage, ongoing pregnancy and live births. Fertilisation was defined as zygotes containing two pronuclei after the ICSI or IMSI procedures. A pregnancy was defined as the presence of a gestational sac with a heartbeat that was visualised by ultrasound 4–6 weeks after embryo transfer. A miscarriage was defined as the termination of the pregnancy up to 20 weeks of gestation. When appropriate, Student’s t-tests, Mann–Whitney tests and Chi-squared tests were utilised, and *P* < 0.05 was considered significant.

## Results

The distributions of the main characteristics of the cycle observed for the ICSI and IMSI procedures were equal (*P* > 0.05). These data are summarised in Table 1.

**Table 1:** Comparison between the ICSI and IMSI procedures

	**Total**		***P***	**Leukocytospermia**		***P***	**Non-leukocytospermia**		***P***
	**ICSI**	**IMSI**		**ICSI**	**IMSI**		**ICSI**	**IMSI**	
Cycles	50	50		25	25		25	25	
Leukocytes in semen (x10^6^)(mean ± SD)	1.5 ± 1.8 (0–8)	2.5 ± 7.5 (0–50)	0.41	2.4 ± 1.7 (1–8)	4.8 ± 10.5(1–50)	0.10	0.4 ± 0.3 (0–0.8)	0.4 ± 0.3 (0–0.8)	0.76
Female age (years)	34.4 ± 3.6	34.8 ± 3.9	0.67	34.4 ± 3.8	34.8 ± 4.1	0.75	34.5 ± 3.6	34.8 ± 3.8	0.79
Male age (years)	36.9 ± 5.0	37.2 ± 5.6	0.80	37.2 ± 5.0	37.4 ± 5.2	0.91	36.7 ± 5.0	37.0 ± 6.0	0.81
Aetiology (%)			0.10			0.43			0.26
Male	48% (24/50)	40% (20/50)		56% (14/25)	36% (9/25)		40% (10/25)	44% (11/25)	
Idiopathic	16% (8/50)	24% (12/50)		16% (4/25)	20% (5/25)		16% (4/25)	28% (7/25)	
Tuboperitoneal	10% (5/50)	16% (8/50)		12% (3/25)	24% (6/25)		8% (2/25)	8% (2/25)	
Endometriosis	18% (9/50)	8% (4/50)		8% (2/25)	4% (1/25)		28% (7/25)	12% (3/25)	
Tuboperitoneal + endometriosis	6% (3/50)	----		4% (1/25)	----		8% (2/25)	---	
Male + tuboperitoneal	2% (1/50)	8% (4/50)		4% (1/25)	8% (2/25)		----	8% (2/25)	
Male + t endometriosis	----	4% (2/50)		----	8% (2/25)			---	
Semen parameters^a^ (mean ± SD)									
Total sperm count (x10^6^/mL)	56.5 ± 49.5	50.9 ± 40.3	0.87	43.2 ± 44.5	41.0 ± 34.8	0.70	69.7 ± 51.5	60.8 ± 43.6	0.58
Motility (% spermatozoa)									
-rapid + slow\progression	43.1 ± 19.7	51.4 ± 22.2	0.09	35.8 ± 17.8	44.3 ± 21.8	0.17	50.7 ±17.9	58.6 ± 20.8	0.22
-non-progressive	11.5 ± 11.4	9.6 ± 11.2	0.08	11.9 ± 12.3	10.5 ± 13.7	0.17	11.2 ± 10.3	8.6 ± 8.3	0.25
-immotile	42.6 ± 20.6	38.6 ± 20.4	0.42	50.4 ± 23.6	45.8 ± 21.0	0.50	35.9 ± 13.6	31.4 ± 17.3	0.36
Morphology									
-normal forms (%)	14 ± 10.5	----		10.1 ± 0.8	----		17.9 ± 20.3	----	
Vitality (%)	46.9 ± 19.2	44.2 ± 19.6	0.66	45.1 ± 17.5	43.1 ± 20.5	0.81	48.8 ± 20.9	45.3 ± 18.7	0.52
MSOME ^b^ (%)									
-normal spermatozoa	----	1.13 ± 1.65		----	0.92 ± 1.43		----	1.35 ± 1.87	
-spermatozoa with vacuoles occupying >50% of the nuclear area	----	35.9 ± 23.05		----	37.8 ± 20.2			34.0 ± 25.9	
Metaphase II oocytes (n)	7.8 ± 4.3	7.8 ± 5.2	0.69	8.5 ± 4.2	8.2 ± 5.7	0.66	7.3 ± 4.2	7.4 ± 4.9	0.81
Fertilisation rate (%)	59.9 ± 28.7	63.5 ± 26.5	0.55	57.9 ± 30.2	67.6 ± 24.6	0.35	61.9 ± 27.7	59.5 ± 28.1	0.86
Embryos transfer (n)	2.1 ± 0.9	2.4 ± 0.8	0.21	2.2 ± 0.9	2.3 ± 0.8	0.90	2.1 ± 1.0	2.6 ± 0.9	0.10
Implantation rate (%)	13.2% (14/106)	17.2% (19/111)	0.54	12.3% (7/54)	17.5% (10/57)	0.68	13.5% (7/52)	16.7% (9/54)	0.84
Pregnancy/cycle (%)	24% (12/50)	26% (13/50)	0.81	24% (6/25)	28% (7/25)	0.74	24% (6/25)	24% (6/25)	1
Miscarriage (%)	0	7.7% (1/13)	0.32	0	14.3% (1/7)	0.33	0	0	----
Ongoing pregnancy/cycle (%)	24% (12/50)	24% (12/50)	1	24% (6/25)	24% (6/25)	1	24% (6/25)	24% (6/25)	1
Live birth/cycle (%)	24% (12/50)	24% (12/50)	1	24% (6/25)	24% (6/25)	1	24% (6/25)	24% (6/25)	1

^a^ Semen quality parameters according to the World Health Organization [1]; and ^b^ motile sperm organelle morphology examination.

### ICSI procedure

Whereas there seems to be an association between an increased number of leukocytes and a decreased quality of semen samples, our data showed that leukocytospermia did not have a negative influence on the fertilisation rate (Group I: 57.9 ± 30.2%, Group II: 61.9 ± 27.7%; *P* = 0.74), implantation rate (Group I: 12.3%, 7/54; Group II: 13.5%, 7/52; *P* = 0.93), clinical pregnancy rate per cycle (Group I: 24%, 6/25; Group II: 24%, 6/25; *P* = 1.0), miscarriage rate (Group I: 0, Group II: 0), ongoing pregnancy rate (Group I: 24%, 6/25; Group II: 24%, 6/25; *P* = 1.0), or live birth per cycle (Group I: 24%, 6/25; Group II: 24%, 6/25; *P* = 1.0) after ICSI. The results and characteristics of the patients are shown in Table 2.

**Table 2:** ICSI Procedure: Comparison between the Leukocytospermia (Group I) and Non-leukocytospermia (Group II) groups

	**ICSI procedure**		
	**Group I Leukocytospermia**	**Group II Non-leukocytospermia**	***P* value**
Cycles	25	25	
Leukocytes in semen (x10^6^) (mean ± SD)	2.4 ± 1.7 (1–8)	<1	
Female age (years)	34.4 ± 3.8	34.5 ± 3.6	>0.99
Male age (years)	37.27 ± 5.0	36.7 ± 5.0	0.71
Aetiology (%)			0.41
Male	56% (14/25)	40% (10/25)	
Idiopathic	16% (4/25)	16% (4/25)	
Tuboperitoneal	12% (3/25)	8% (2/25)	
Endometriosis	8% (2/25)	28% (7/25)	
Tuboperitoneal + endometriosis	4% (1/25)	8% (2/25)	
Male + tuboperitoneal	4% (1/25)	----	
Semen parameters^a^ (mean ± SD)			
Total sperm count (x10^6^/mL)	43.2 ± 44.5	69.7 ± 51.5	0.06
Motility (% spermatozoa)	35.8 ± 17.8	50.7 ± 17.9	0.005
-rapid + slow progression	11.9 ± 12.3	11.2 ± 10.3	0.72
-non-progressive	50.4 ± 23.6	35.9 ± 13.6	0.0069
-immotile Morphology			0.06
-normal forms (%)	10.1 ± 0.8	17.9 ± 20.3	0.68
Vitality (%)	45.1 ± 17.5	48.8 ± 20.9	0.72
Metaphase II oocytes (n)	8.5 ± 4.2	7.3 ± 4.2	0.32
Fertilisation rate (%)	57.9 ± 30.2	61.9 ± 27.7	0.74
Embryos transfer (n)	2.2 ± 0.9	2.1 ± 1.0	0.60
Implantation rate (%)	12.3% (7/54)	13.5% (7/52)	0.93
Pregnancy/cycle (%)	24% (6/25)	24% (6/25)	1
Miscarriage (%)	0	0	
Ongoing pregnancy/cycle (%)	24% (6/25)	24% (6/25)	1
Live birth/cycle (%)	24% (6/25)	24% (6/25)	1

^a^ Semen quality parameters according to the World Health Organization [1].

### IMSI procedure

Similar to our ICSI results, we identified an association between an increased number of leukocytes and decreased quality of semen samples. However, our data showed that leukocytospermia did not have a negative influence on the fertilisation rate (Group I: 67.6 ± 24.6%, Group II: 59.5 ± 28.1%; *P* = 0.36), implantation rate (Group I: 17.5%, 10/57; Group II: 16.7%, 9/54; *P* = 0.90), clinical pregnancy rate per cycle (Group I: 28%, 7/25; Group II: 24%, 6/25; *P* = 1.0), miscarriage rate (Group I: 14.3%, 1/7; Group II: 0; *P* = 0.33), ongoing pregnancy rate (Group I: 24%, 6/25; Group II: 24%, 6/25; *P* = 1.0), or live births per cycle (Group I: 24%, 6/25; Group II: 24%, 6/25; *P* = 1.0) after an IMSI. These results are summarised in Table 3.

**Table 3:** IMSI Procedure: Comparison between the Leukocytospermia (Group I) and Non- leukocytospermia (Group II) groups

	**IMSI procedure**		
	**Group I Leukocytospermia**	**Group II Non-leukocytospermia**	***P* value**
Cycles	25	25	
Leukocytes in semen (x10^6^) (mean ± SD)	4.8 ± 10.5 (1–50)	<1	
Female age (years)	34.8 ± 4.1	34.8 ± 3.8	>0.99
Male age (years)	37.4 ± 5.2	37.0 ± 6.0	0.84
Aetiology (%)			0.35
Male	36% (9/25)	44% (11/25)	
Male age (years)	37.2 ± 5.0	36.7 ± 5.0	0.71
Idiopathic	20% (5/25)	28% (7/25)	
Tuboperitoneal	24% (6/25)	8% (2/25)	
Endometriosis	4% (1/25)	12% (3/25)	
Male + endometriosis	8% (2/25)	---	
Male + tuboperitoneal	8% (2/25)	8% (2/25)	
Semen parameters^a^ (mean ± SD)			
Total sperm count (x10^6^/mL)	41.0 ± 34.8	60.8 ± 43.6	0.068
Motility (% spermatozoa)			0.005
-rapid + slow progression	44.3 ± 21.8	58.6 ± 20.8	0.01
-non-progressive	10.5 ± 13.7	8.6 ± 8.3	0.82
-immotile	45.8 ± 21.0	31.4 ± 17.3	0.0048
Vitality (%)	43.1 ± 20.5	45.3 ± 18.7	0.72
MSOME b (%)			
-normal spermatozoa	0.92 ± 1.43	1.35 ± 1.87	0.43
-spermatozoa with vacuoles occupying >50% of the nuclear area	37.8 ± 20.2	34 ± 25.9	0.22
Metaphase II oocytes (n)	8.2 ±5.7	7.4 ± 4.9	0.65
Fertilisation rate (%)	67.6 ± 24.6	59.5 ± 28.1	0.36
Embryos transfer (n)	2.3 ± 0.8	2.6 ± 0.9	0.28
Implantation rate (%)	17.5% (10/57)	16.7% (9/54)	0.90
Pregnancy/cycle (%)	28% (7/25)	24% (6/25)	0.74
Miscarriage (%)	14.3% (1/7)	0	0.33
Ongoing pregnancy/cycle (%)	24% (6/25)	24% (6/25)	1
Live birth/cycle (%)	24% (6/25)	24% (6/25)	1

^a^ Semen quality parameters according to the World Health Organization [1]; and ^b^ the motile sperm organelle morphology examination.

## Discussion

The goal of the ICSI and IMSI cycles is the transfer of high-quality embryos because it is well known that the implantation rates are directly related to the capacity of the embryo to interact with a receptive endometrium. Given that embryo quality depends on the quality of both gametes, it is important to assess the condition of the male gametes by analysing seminal characteristics. To evaluate male factors, the WHO manual established that parameters such as sperm concentration, motility, viability, morphology and leukocyte count should be assessed [1]. *In vitro* studies have shown that a high level of leukocytes can induce oxidative stress and alter sperm parameters. However, the *in vivo* effects of leukocytes are less clear. Although some authors have reported an association between leukocytospermia and alterations in sperm functions [4,5,7,9,33], a positive effect on the morphology of leukocytes has also been described [3,35]. Additionally, some studies have reported that in infertile individuals, leukocytes do not affect sperm function [36] and are associated with increased pregnancy rates after assisted reproduction cycles compared to infertile patients without leukocytospermia [17]. Thus, the significance of leukocytospermia remains controversial, and its relevance to male infertility has prevented questions such as “Seminal leukocytes: passengers, terrorists or good Samaritans?” [37] and “Semen leukocytes: friends or foes?” [3] from being answered for almost two decades.

With regard to leukocytospermia, the first issue to be considered is that there is no consensus regarding the threshold for the number of white blood cells in seminal samples. Wolff [38] considered the value proposed by the WHO to be too low, whereas Sharma et al. [39] and Punab et al. [40] postulated that 1.10^6^ leukocytes could be an inadequately high threshold. These discrepancies seem to be due to the different end points evaluated in these investigations [41]. Moreover, there seems to be a considerable amount of spontaneous variation in white blood cell counts in infertile men, which can reach a spontaneous downward variation of up to 43% [42].

It has been determined that neutrophils and macrophages constitute 95% of seminal leukocytes, which may promote male subfertility by damaging sperm via the generation of ROS and the induction of apoptosis [43]. In semen samples associated with leukocytospermia, DNA damage is promoted in a cascade-like manner, which suggests that ROS play a major role in these alterations [44]. Furthermore, an elevated leukocyte count, in addition to supplying the main source of ROS in the seminal plasma, may also be involved in retrovirus transmission [45]. It is postulated that ROS may damage the sperm membrane, reduce sperm motility and capacity for fertilisation, and compromise sperm DNA [46]. However, the significance of the association between leukocytospermia and ROS must be further clarified.

Although it seems clear that a high production of ROS is associated with male infertility and that leukocytes are the predominant source of ROS in sperm suspensions, there is doubt as to whether these leukocytes infiltrate the epididymis and are thus responsible for the impaired function of the semen [47]. Recently, Ramya et al. [48] have reported that in men with normal sperm concentrations, sperm dysfunction may occur as the result of the cytotoxic effects of nitric oxide (NO). NO is a free radical that is believed to be a mediator of sperm function [49], and leukocytospermia is likely responsible for the increase in NO concentrations. Nevertheless, when patients with leukocytospermia show increased activity of NO synthase with normal levels of NO, sperm motility and viability are not compromised [48].

Another point for consideration is whether a leukocytospermic sample is of sufficiently good quality based on all of the other parameters evaluated. Erenpreiss et al. [44] reported that alterations of DNA integrity are significant only between semen samples of very good and very poor quality, and they stress that the concentration and motility parameters of normal semen may be resistant to the DNA damage associated with leukocytospermia. Even when considering that leukocytospermia is associated to the production of ROS and impairs semen function, it remains unclear whether this seminal dysfunction would have any impact on ART outcomes. Although lower fertilisation and cleavage rates have been reported in the presence of leukocytospermia [15,50], a more recent investigation reported that the ICSI outcomes were impaired when seminal samples associated with this condition were employed, even though there was a trend for an association with male infertility [16,17]. Furthermore, it was demonstrated that fertile semen donors could also have leukocytospermia (≥10^6^/mL) [51]. Another study showed a negative effect of ROS on conventional IVF cycles, which was not observed for ICSI cycles [52]. Alternatively, the impact of ROS in ART cycles may be more complex than simply their presence or absence in the semen samples [16,53], given that ROS seem to have distinct effects on semen. Therefore, ROS may additionally exert a beneficial influence in the process of capacitation [54-57].

The results of our investigation demonstrated a trend towards association between leukocytospermia with important semen parameters, including concentration and motility, which were reduced in comparison with control patients. This finding is in contrast with the observations of Lackner et al. [16], who did not find any influence of leukocytes on sperm motility. However, our observations are in agreement with other previous reports. Fedder [4] observed an association between high concentrations of certain white blood cell products and the inhibition of experimentally measured sperm functions (e.g., motility). Arata de Bellabarba et al. [5] reported that leukocytospermia occurs frequently in infertile patients and that it is associated with poor semen quality parameters. Saleh et al. [7] found that leukocytospermia was associated with a significant decrease in sperm motility. Aziz et al. [33] reported that the leukocytic concentration in semen was significantly and negatively correlated with the motile sperm percentage but not with sperm concentration. The results of Moskovtsev et al. [9] indicated that leukocytospermia has a significant negative effect on the standard semen parameters of concentration, motility, and morphology. In addition, Barraud-Lange et al. [17] analysed 1955 semen samples and found a significant correlation between the amount of leukocytes and the deterioration of semen parameters (e.g., sperm count, total sperm number and motility). Thus, our data emphasise the correlation between leukocytospermia and abnormal semen quality. Conversely, it should be noted that the correlation between the morphology of the semen and the presence of leukocytes was a contradictory point among the previous studies. The existence of a leukocyte function in the elimination of abnormal spermatozoa from ejaculated semen has already been analysed [3]. In our study, although the average numbers of sperm with a normal form were always lower in the groups with leukocytospermia, there was no significant difference from the samples without leukocytospermia. This finding is consistent with other previously published studies [17].

With regard to the fertilisation, implantation, miscarriage, ongoing pregnancy and live birth rates, we did not observe any statistically significant differences between the groups with or without an increased number of leukocytes in the seminal fluid. Our data are in agreement with the report of Lackner et al. [16], who found that leukocytospermia might not have a negative effect on the outcomes of IVF/ICSI cycles. We can postulate that the techniques of ICSI and IMSI are capable of bypassing the negative effects of leukocytospermia and the consequences of ROS on the sperm quality. Moreover, our group routinely performs IMSI to avoid insemination of oocytes with sperm that have large nuclear vacuoles (LNV; spermatozoa with vacuoles occupying >50% of the nuclear area), which reflects the presence of abnormal chromatin packaging, thereby facilitating sperm DNA damage [58]. The selection of sperm without LNV may prevent the injection of sperm with DNA fragmentation, which, according to Fariello et al. [10], is likely to occur in samples with leukocytospermia. However, the possibility that the presence of leukocytes did not have a deleterious effect on ART outcomes cannot be eliminated. Barraud-Lange et al. [17] in fact proposed that leukocytospermia is protective *in vivo* and that it is associated with normal or improved IVF and ICSI results when compared with non-leukocytic patients with infertility. In a study analysing 3508 IVF/ICSI cycles, the fertilisation rate, cleavage rate, clinical pregnancy rate, gestational age, and mean infant weight were significantly improved when seminal leukocytes were present, regardless of the technique used. The only negative side effects associated with a high level of seminal leukocytes (≥10^6^/mL) were an elevated rate of early pregnancy loss (from 26.6% to 40.5%) and a 3-fold increase in the percentage of ectopic pregnancies. Thus, further trials with larger sample sizes will be helpful in determining the *in vivo* role of seminal leukocytes.

A limitation of our study is the low number of sperm samples and cycles that were analysed. The present study failed to show any statistically significant differences in the clinical outcomes, including clinical pregnancy, ongoing pregnancy and live birth rates. This observation may be related to the small cumulative sample size (i.e., insufficient power). With respect to ongoing pregnancy rates, based on the results obtained with the leukocytospermia and non-leukocytospermia groups in ICSI cycles (24%, 6/25 and 24%, 6/25, respectively) and in IMSI cycles (24%, 6/25 and 24%, 6/25, respectively), the ability to detect a difference of 5% with a power of 80% and to reach definitive conclusions would require more than 4000 cycles. Thus, future controlled trials that provide more information on clinical parameters will help to clarify the reliability of these results. Alternatively, it should be emphasised that other published studies reported data from small samples of leukocytospermic men. Lackner et al. [16] evaluated 20 men with leukocytospermia in groups of 75 individuals. During a 7-year follow-up in an ART programme, a study reported a concentration of ≥10^6^/mL leukocytes in the semen of only 73 men out of a total of 1955 who were analysed [17]. Therefore, despite our small study population, we believe that our work can contribute to the understanding of leukocytospermia.

In conclusion, the results of this investigation indicate that leukocytospermia may not have a negative effect on the outcomes of ICSI and IMSI cycles. Nevertheless, it is necessary to more precisely determine the effects, if any, of seminal leukocytes on the fertilisation and implantation processes and to establish a more reliable leukocyte threshold that could eventually demonstrate negative influences on the ART procedures.

## Competing interests

The authors declare that they have no competing interests.

## Authors’ contributions

MC designed and coordinated the study. All of the authors were responsible for the data collection, analysis and interpretations presented in the manuscript. MC, JBAO, RLRB and JG performed the statistical analyses and wrote the manuscript. JG reviewed the manuscript. All authors read and approved the final manuscript.
